# OppA, the ecto-ATPase of *Mycoplasma hominis *induces ATP release and cell death in HeLa cells

**DOI:** 10.1186/1471-2180-8-55

**Published:** 2008-04-04

**Authors:** Miriam Hopfe, Birgit Henrich

**Affiliations:** 1 Institute of Medical Microbiology and Center for Biological Medical Research, Heinrich-Heine-University, Moorenstrasse 5, 40225 Duesseldorf, Germany

## Abstract

**Background:**

In the facultative human pathogen *Mycoplasma hominis*, which belongs to the cell wall-less *Mollicutes*, the surface-localised substrate-binding domain OppA of the oligopeptide permease was characterised as the main ecto-ATPase.

**Results:**

With the idea that extra-cellular ATP could only be provided by the infected host cells we analysed the ATP release of HeLa cells after incubation with different preparations of *Mycoplasma hominis*: intact bacterial cells, the membrane fraction with or without OppA, recombinant OppA as well as an ATPase-deficient OppA mutant. Release of ATP into the supernatant of the HeLa cells was primarily determined in all samples lacking ecto-ATPase activity of OppA. In the presence of the ATPase inhibitor DIDS the amount of ATP in the OppA-containing samples increased. This increase was maximal after incubation with fractions containing OppA protein indicating that OppA is involved in ATP release and subsequent hydrolysis. Real-time PCR analyses revealed that the proliferation of HeLa cells is reduced after infection with *M. hominis *and flow cytometry experiments established that OppA induces greater apoptosis than necrosis of HeLa cells whereas the preservation of ecto-ATPase activity of OppA induces apoptosis.

**Conclusion:**

The OppA induced ATP-release and -hydrolysis induced cell death of *M. hominis *infected HeLa cells was predominantly due to apoptosis rather than necrosis. Future work will elucidate whether the induction of apoptosis is indispensable for survival of these non-invasive pathogen.

## Background

In contrast to common belief, nucleotides can be found in significant concentrations outside cells [1]. Nucleotides, such as ATP, ADP, UTP and UDP, and a variety of di-adenosine polyphosphates act as extra-cellular signalling substances in virtually all tissues. Extra-cellular ATP has profound effects on cellular functions: causing plasma membrane depolarisation, Ca^2+ ^influx, and cell death [2,3]. The identification of two families of nucleotide receptors (P2 receptors) enabled molecular analyses of nucleotide signalling [4,5]. Nucleotides released to the extra-cellular medium may exert their effects on other cells in the vicinity of the secretion site and modulate biological processes by binding to specific cell surface receptors [6].

The signalling of nucleotides is terminated by enzymes on the extra-cellular surface which sequentially degrade nucleoside 5'-triphosphate to their respective nucleosides and free phosphate or pyrophosphate. Adenosine itself can modulate cellular functions (e.g. apoptosis) via specific adenosine receptors [7] or enter the purine salvage after re-uptake by plasma membrane-located adenosine transporters [8]. Within the last decade enzymes that have the potential to hydrolyse extra-cellular nucleotides have been characterised in detail. ATPase activity was found in association with various cell types in the circulating system, nervous and other tissues and shown to impact on several patho-pysiological processes [9]. Besides the members of the ecto-nucleoside triphosphate diphosphohydrolases (E-NTPDase)-family found in eukaryotes, other ecto-ATPases have been characterised in both eukaryotes and prokaryotes, such as in streptococci [10] and in several protozoan parasites including the genera *Toxoplasma *[11], *Leishmania *[12-14], *Entamoeba *[15], *Trichomonas *[16], *Crithidia *[17] and *Trypanosoma *[18-20]. Common to all was the ecto-ATPase activity shown to be dependent on divalent cations and to be inhibited by the impermeant ATPase-inhibitor 4', 4', diisothiocyanostilbene 2'2'disulfonic acid (DIDS) [15] and suramin (an antagonist of P_2 _receptors and some ecto-ATPases) [21].

The ecto-ATPases and cell surface NTPDases hydrolyse all nucleoside triphosphates at the external surface of the cell membranes [22,23] whereas the differences in their ability to hydrolyse nucleoside diphosphates, the modes of anchoring to membranes and their distribution in tissues led to a grouping into distinct sub-families [23]. ATP can also be hydrolysed by other specific ATPases (F-, P- or V-type ATPases) or by nonspecific alkaline phosphatases. Whereas these ATPases expose an internal ATP binding site, thus not fulfilling the requirements of an ecto-ATPase, external ecto-phosphatase activities have been described eg. in malpighian tubules of *Rhodnius prolixus *[24], *Leishmania amazonensis *[25] and *Trypanosoma cruzi *[26].

Several hypotheses for the function of ecto-ATPases in various cell types have been proposed: (i) protection from the cytolytic effect of extra-cellular ATP [3,27], (ii) regulation of ecto-kinase substrate concentration [22], (iii) involvement in signal transduction [28,29], and (iv) involvement in cellular adhesion [30,31]. Extra-cellular ATP was shown to be important in the activation of macrophage surface-associated purine receptors and subsequent macrophage cell death, whereas in the presence of an ecto-ATPase activity the ATP-induced cell death was inhibited [32]. Cell death can either be the consequence of a passive, degenerative process termed necrosis or the result of an active process leading to apoptosis. When a cell dies by necrosis the first changes occur on the plasma membrane with signs of progressive discontinuity that cause general cell hydration and swelling as well as organelle disruption. In the case of apoptotic cell death the nucleus appears greatly altered with a diffuse interchromatin distribution compared to the normal perinuclear and perinucleolar dense heterochomatin pattern. Plasma membrane and organelles are preserved longest which characterises some apoptotic models. The most common mechanism of apoptotic cell removal *in vivo *is by phagocytosis, however it has been reported *in vitro *that some apoptotic cells undergo a late process of secondary necrosis. Analysing the role of apoptosis in bacterial pathogenesis three pathogenic strategies appear to be involved in programmed cell death:

i.) Activation of apoptosis to destroy host cells: Bacteria such as *Corynebacterium diphtheriae*, *Pseudomonas *spp, *Actinobacillus actionomycetemcomitans *and *Bacillus anthracis *produce exotoxins which affect killing of macrophages and thus protect against phagocytosis.

ii.) Utilisation of apoptosis to initiate inflammation: IL-1β, the major pro-inflammatory cytokine is mainly produced by macrophages. Its expression is enhanced in apoptotic macrophages.

iii.) Inhibition of host cell apoptosis: Several pathogens, including herpes-, pox-, and baculoviridae have been shown to inhibit host cell apoptosis. This is beneficial for survival of intracellular pathogens.

In *Mycoplasma hominis*, a cell wall-less bacterium colonising the human uro-genital tract, we have identified a cytoadhesive 100 kDa lipoprotein as OppA, the surface-exposed substrate-binding domain of an oligopeptide permease [33,34]. Computer analysis revealed an ATP-binding loop in the C-terminal region of the polypeptide chain consisting of Walker A and B motifs. ATP-binding was confirmed by ATP-affinity chromatography and tryptic digestion of OppA was protected by ATP and ADP but not by GTP or CTP. We concluded that OppA binds ATP and ADP but not GTP or CTP. The detection of ATPase activity on the surface of *M. hominis *and the comparative analysis of equimolar amounts of OppA in intact mycoplasma cells and in its purified form showed that more than 80 % of the surface-localised ATPase activity of *M. hominis *is derived from OppA, implicating that OppA is the main ATPase on the surface of mycoplasma cells [35].

Membrane interactions between the surface of mycoplasma and their host are of critical importance for colonisation and nutrition-up-take and therewith the survival of the bacteria. The results presented in this study provide evidence that OppA, the main *ecto*ATPase of *M. hominis *is involved in processes that induces ATP release from and damage of the host cell.

## Results

### Induction of ATP release from HeLa cells by OppA

**Figure 1:**
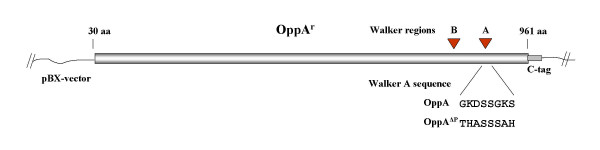
**Scheme of the OppA expressing vectors**. The OppA and OppA^ΔP-loop^-expressing regions of the pBX-vectors are schematically shown with declaration of the original and mutated Walker A region.

**Figure 2:**
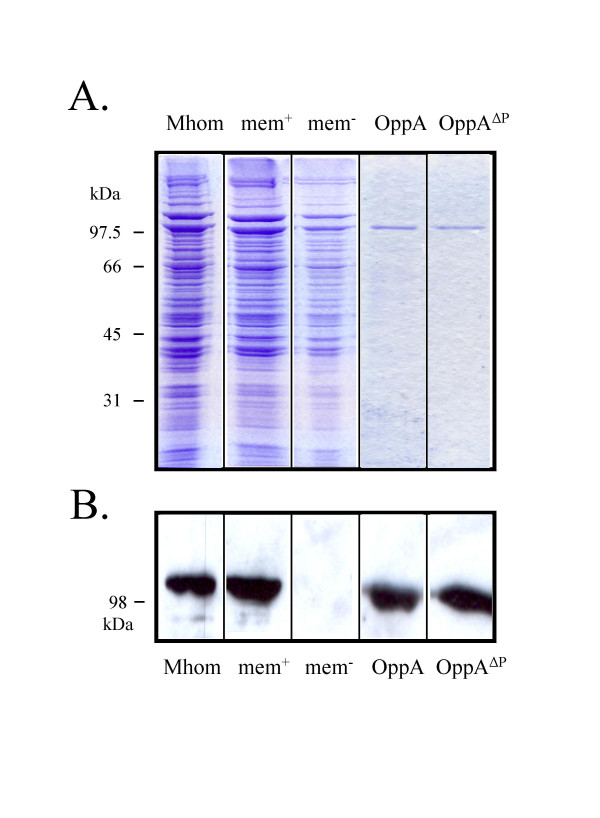
**Protein preparations used in the infection assay**. A: The different mycoplasma samples; *M. hominis *(Mhom), membrane proteins (mem+), membrane proteins without OppA (mem-), purified OppA and OppA^ΔP-loop^-mutant; corresponding to 0,5 μg/ml OppA, were separated by SDS-PAGE and subsequently stained with Coomassie Blue. B: Westernblot analysis of the protein samples immunostained with the OppA specific monoclonal antibody BG11.

**Figure 3:**
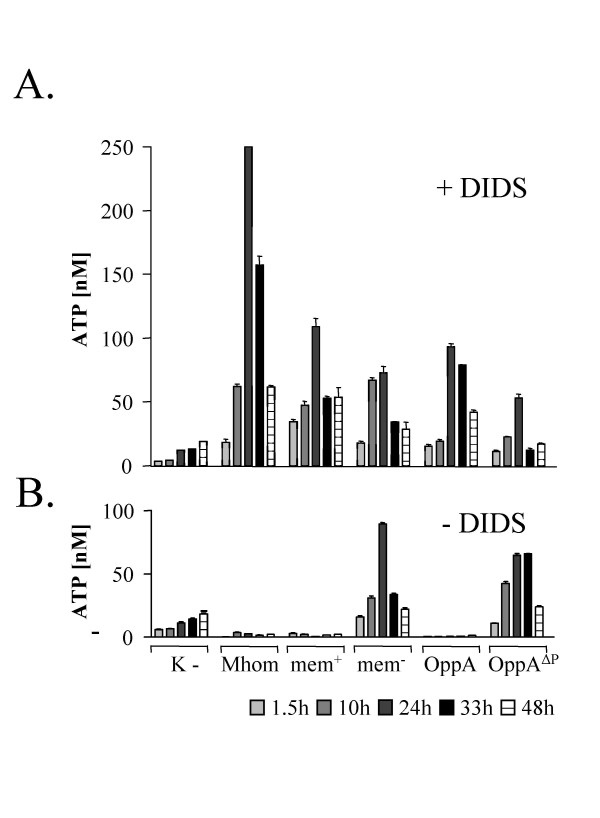
**Release of ATP from *M. hominis *infected HeLa cells**. HeLa cells were incubated with *M. hominis *(Mhom), membrane proteins (mem+), membrane proteins without OppA (mem-), purified OppA and OppA^ΔP-loop^-mutant for 1.5 to 48 h as described in the Material and Methods section. Infection assays were done in the presence (A) or absence (B) of 500 μM ATPase inhibitor 4',4', diisothiocyanostilbene 2'2'disulfonic acid (DIDS). The ATP concentration in the culture supernatants was measured using a luciferase assay (ATP Determination Kit). Results are expressed as means ± SD of triplicates.

OppA was characterised as the main ecto ATPase of *M. hominis*. Presuming that external ATP could only be provided by the infected host cells we analysed the effect of *M. hominis *cells, as well as different components of the cell membrane, on the release of ATP from HeLa cells. HeLa cells were incubated with (i) intact *M. hominis *cells, (ii) the membrane protein fraction with or (iii) without OppA, (iv) the purified OppA protein and (v) OppA^ΔP-loop^, an OppA variant with a mutation in the region of the Walker A motif and therefore deficient ATPase activity (Fig 1; [35]). The protein pattern of the mycoplasma protein samples is shown in Coomassie Blue staining (Fig. 2A) and the presence or absence of OppA in Westernblot analysis using the OppA specific monoclonal antibody BG11 (Fig. 2B). The medium was supplemented with the ATPase inhibitor 4', 4', diisothiocyano-stilbene 2'2'disulfonic acid (DIDS) to prevent degradation of ATP (Fig. 3A). In the supernatant of uninfected HeLa cells the ATP content was not altered by the ATPase inhibitor DIDS (Fig. 3A/B; [K-]). Independent of the used protein sample, the ATP concentration in the supernatant of the HeLa cells reached a maximum after 24 hours. A 19- and 8-fold increase was measured after incubation with *M. hominis *cells and the membrane fraction (mem^+^), respectively. The membrane proteins showed nearly the same result as the purified OppA protein whereas incubation with the OppA-depleted membrane fraction resulted in a 25 % decrease of ATP release. Nevertheless, the OppA-depleted membrane fraction and the OppA mutant (OppA^ΔP-loop^) which are unable to hydrolyse ATP, still led to a release of ATP from the HeLa cells. This finding suggested that OppA induces an ATP release from HeLa cells and that in addition other, so far undefined components of the membrane fraction also lead to ATP secretion.

In contrast, in the absence of an ATPase inhibitor, ATP degradation was apparent in all supernatants of HeLa cells incubated with OppA-containing proteins (mycoplasma cells, the OppA containing membrane protein fraction (mem+) and the purified OppA) whereas the incubation with the OppA-depleted membrane protein fraction or the OppA mutant resulted in a 5- to 6-fold increase of ATP in supernatant.

These data provide evidence that *M. hominis *induces ATP release from HeLa cells and that OppA is not only involved in this process but in the subsequent ATP hydrolysis.

### *Mycoplasma. hominis *inhibited the growth of HeLa cells

**Figure 4:**
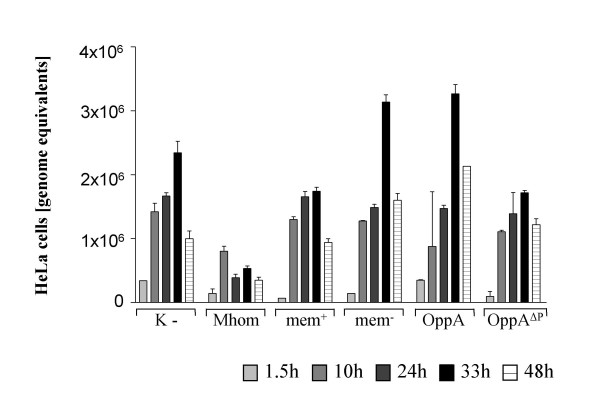
**TaqMan PCR to quantify HeLa cell counts**. The HeLa cell counts of an infection assay after incubation with *M. hominis *(Mhom), membrane proteins with (mem^+^) or without OppA (mem^-^), purified OppA and OppA^ΔP-loop^-mutant were calculated by quantifying the copy numbers of the GAPDH-gene using real-time PCR.

Next, we analysed the effect of *M. hominis *on the growth of HeLa cells by amplifying a part of the house keeping gene glyceraldehyde 3-phosphate dehydrogenase (GAPDH) with real-time PCR as a quantitative measurement of HeLa cells. As depicted in Figure 4 the number of uninfected HeLa cells or HeLa cells incubated with the *M. hominis *membrane fraction (mem^+^), purified OppA (OppA) or the purified OppA mutant (OppA^ΔP-loop^) increased up to 72 h and then declined, whereas in HeLa cells infected with *M. hominis*, the numbers barely increased for 24 h before declining. These findings suggested that an *M. hominis *infection has an inhibitory effect on the growth of HeLa cells, whereas the protein preparations have no sustainable effect. Thus, we decided to characterise the early event of infection and responses to the different protein samples in detail by determining the cell death of HeLa cells.

### OppA-induced cell death

We analysed HeLa cells in the described infection assays by flow cytometry for markers of apoptosis and necrosis. Using Annexin-V-FITC which binds to negatively charged phospholipids such as phosphatidylserine, these early markers of apoptosis were detected. The vital dye 7-amino-actinomysin (7AAD) was used as a marker for necrotic cells as it binds to nucleic acids that are only accessible when membrane integrity is lost such as occurs in necrosis or the later stages of apoptosis.

**Figure 5:**
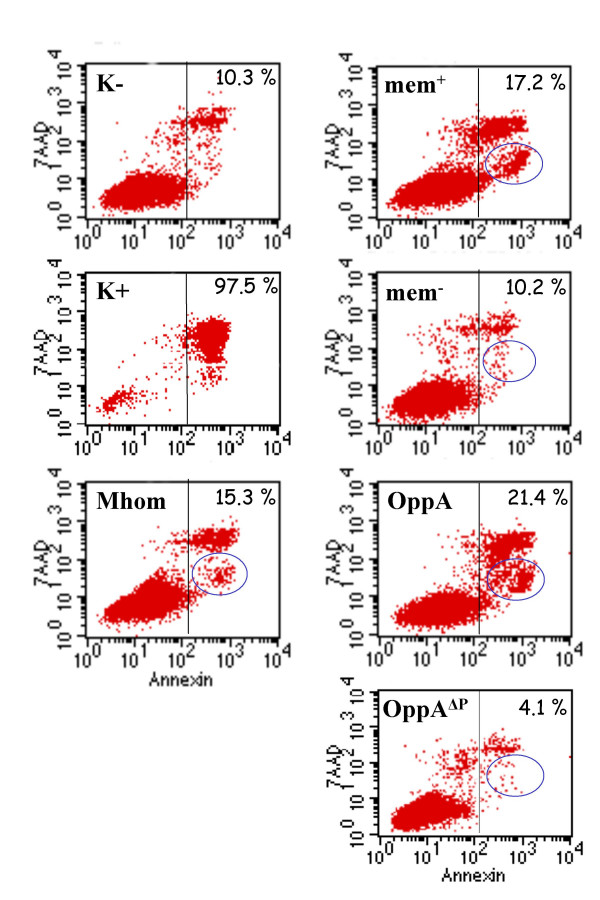
**Flow cytometric analysis**. Flow cytometric analysis of (i) uninfected HeLa cells (K-), (ii) HeLa cells after UV irradiation (K+), (iii) incubation with *M. hominis *cells (Mhom), (iv) mycoplasmal membrane protein fraction (mem^+^), (v) membrane proteins without OppA (mem^-^); (vi) purified OppA protein (OppA) or (iv) OppA^ΔP-loop^-mutant. HeLa cells (1 × 10^6^/ml) were incubated at 37°C for 2h with 1 μg/ml OppA, then stained with Annexin-V-FITC and 7-AAD and subjected to flow cytometric analysis. Necrotic cells were Annexin-V-FITC (+) and 7AAD (+), whereas the apoptotic cells (marked by circle ) were Annexin-V-FITC (+) and 7AAD (-).

As shown in Figure 5 the proportion of dead cells in HeLa cultures stimulated with *M. hominis *(Mhom) and membrane fraction (mem^+^) was approximately 15 % and 17 %, respectively. In HeLa cells stimulated with the OppA-depleted membrane fraction (mem^-^) or the OppA^ΔP-loop^-mutant, the quantity of dead cells corresponded to those found in the uninfected negative control (K-). Interestingly, the proportion of dead cells after incubation with purified OppA increased up to 21 %.

Having a closer look at the dead HeLa cells, two distinct Annexin-V-FITC positive populations became obvious, one with a high 7AAD-density corresponding to the UV-irradiated necrotic cells (K+) and one with a low 7AAD-staining that most likely represent the apoptotic cells. As encircled in Figure 5, the apoptotic cells only appeared in those samples containing OppA with ecto-ATPase activity, whereas incubation with the OppA-depleted membrane fraction (mem^-^) or the OppA^ΔP-loop^-mutant resulted in only minimal apoptosis.

These results demonstrate that OppA induces both apoptotic and necrotic cell death in HeLa cells whereas the preservation of ecto-ATPase activity promotes apoptosis.

## Discussion

For a long time, ATP was only regarded as the principal molecule responsible for energy storage inside the cells. In the last decade it has also been detected in nanomolar concentrations in the extra-cellular space where it serves as a signalling molecule. ATP is now known to be released from a variety of eukaryotic cells including tumour cells [36], neutrophils, degranulated platelets [1] and epithelial cells colonised by bacteria [37], as well as in virtually all tissues under conditions of hypoxia [38], ischemia, inflammation [39] and cell necrosis [36].

Although much is known about eukaryote purinergic receptors that bind released nucleotides and NTPDases that hydrolyse released nucleotides, there are only few descriptions of the molecules which influence or induce the release of NTP, especially ATP. Endotoxin was shown not to induce ATP release from a murine macrophage cell line [40] whereas interleukin 1 beta (IL-1β) has been reported to evoke ATP and adenosine release from rat hippocampal slices [41]. ATP itself induces ATP release from astrocytes [42] and endothelium [43]. Our studies provide evidence that OppA, the main ecto-ATPase of *M. hominis*, induces ATP-release from HeLa cells followed by subsequent hydrolysis due to its intrinsic ATPase activity. Using OppA-deficient mycoplasma samples, the ATP release was reduced but not completely inhibited, indicating that, in addition, other (membrane) proteins may affect ATP-liberation. With our previous findings of an additional ATPase activity which was not inhibited by DIDS these data suggest that the ATP concentration released from the HeLa cells may be higher than that measured in the ATP-assay by the use of DIDS [35]. This is to our knowledge the first description that a surface-localised protein of *Mycoplasma *acts as an effector molecule for ATP release from the host.

In contrast to the F- and P-type ATPases which both act intra-cellularly the ATP-binding site of OppA was shown to be extra-cellularly located as in E-type NTPDases. Like E-NTPDases OppA requires Ca^2+ ^or Mg^2+ ^ions for maximal activity to hydrolyse nucleoside triphosphates and, with a subtype-specific potency, also nucleoside diphosphates [9]. In contrast to OppA, which carries a P-loop structure in the catalytic region, all NTPDases share five conserved domains, called apyrase conserved region (ACR), that are involved in the catalytic cycle. These findings indicate that OppA is a member of a different ATPase family.

Degradation of the extra-cellular ATP to adenosine is known to inhibit the growth of several types of cells [44,45] and even to induce apoptosis [46]. The degradation products of ATP normally comprise ADP, AMP and adenosine which are also known as components of the extra-cellular milieu that stimulate purinergic receptors [22,9]. In many cell types the stimulation of the receptors leads to elevation of intracellular Ca^2+ ^[47] and a variety of physiological responses such as activation of caspases, release of cytochrome C and apoptosis [48].

The data presented here demonstrate that *Mycoplasma hominis *inhibits the growth of HeLa cells up to 72 h post infection. The findings that neither the membrane fraction, the purified OppA protein nor the OppA mutant OppA^ΔP-loop ^seem to have a sustainable effect on the growth of HeLa cells, may be due to degradation of the mycoplasma proteins or dilution in relation to the proliferating HeLa cells. By monitoring the cells within the first 24 hours post infection by FACS we detected that the ecto-ATPase activity of OppA provokes apoptosis of the host cells.

These findings are in accordance with the recently published work of Zhang and Lo who showed that *M. hominis *and *M. salivarium*, both surface-colonising species, accelerated apoptosis of 32 D cells and inhibited proliferation, whereas the invasive mollicutes *M. fermentans *and *M. penetrans *not only prevented 32D cell apoptosis but stimulated cell proliferation [49]. A prolonged infection with *M. fermentans *or *M. penetrans *infection for up to 5 weeks induced malignant transformation of the 32D cells [50,51]. Additionally, Gerlic and coworker reported in 2004 that vital *M. fermentans *cells protect against rather than induce apoptosis [52]. Secreted ATP-utilizing enzymes of *Mycobacterium bovis *prevent an ATP-induced macrophage cell death [32] and also *Lactobacillus rhamnosus *secretes two proteins that regulate intestinal epithelial cell anti-apoptotic responses and proliferation [53]. By contrast *Pseudomonas aeruginosa*-secreted products provoke macrophage-killing [54]. To date we do not really know why some bacteria have an apoptotic effect whereas others inhibit apoptosis, however one should bear in mind that the prevention of apoptosis of the host cell would be especially beneficial for intracellular pathogens. Indeed, *M. hominis*, an extra-cellular bacterium provokes apoptosis whereas *M. fermentans*, which is facultatively intra-cellular, inhibits apoptosis. The potential of invasive enteric pathogens to mediate cell death differs. Compared to the invasive *Salmonella *and *Shigella *spp., the extra-cellular bacterium *Escherichia coli *was much weaker in its induction of cell death. Monack and coworker showed that *Salmonella typhimurium *was able to kill between 70 to 90 % of the targeted macrophages, whereas *E. coli *killed only 50 % of the host cells [55]. *Shigella flexneri *invaded HeLa cells but did not cause apoptosis in these cells.

The ability of the *M. hominis *ecto-ATPase OppA to induce the release of ATP from the host and to hydrolyse ATP which ultimately leads to the induction of apoptosis correlates well with the hypothesis of an extra-cellular colonizing pathogen enhancing apoptotic cell death.

There are many differences between apoptosis and necrosis. Only single cells are affected by apoptosis whereas groups of neighbouring cells undergo necrosis. The plasma membrane of apoptotic cells maintains structural continuity in contrast to the plasma membrane of necrotic cells which shows an early lysis. For *M. hominis*, as an extra-cellular parasite, the attachment to the host cell is very important for survival. Thus the induction of apoptosis is less critical for the parasite as firstly, the single, apoptotic cell dies which did not influence the neighbouring cells and secondly, the surface of an apoptotic cell remains intact for the attached mycoplasma enabling the indispensable uptake of nutritional substances from the host cell. In preliminary studies we demonstrated that a colonisation with *M. hominis *induced the entry of Lucifer yellow dye in HeLa cells which can only occur in the event of increased membrane permeability (data not shown). Steinberg described in 1987 that ATP induces the formation of plasma membrane pores that allow the influx and efflux of larger molecules [56]. Further studies aim to elucidate the molecular mechanism by which *M. hominis *and especially OppA mediate host cell death and whether ATP hydrolysis facilitates the nutritional uptake of the oligopeptide permease for bacterial consumption [34] or mere serves as an inducer of apoptosis by regulating the extra-cellular ATP concentration. Many studies have demonstrated that extra-cellular ATP interacts with the P_2 _purinergic receptors [57-59]. At high concentrations ATP induces apoptosis through ligation of the P_2_X_7 _and P_2_Y_1 _receptors and conversely at lower concentrations it provokes cell proliferation suggested by its action via the P_2_Y_2 _receptors [60].

Not only ATP but also various ionic forms of ATP, such as ATP^4- ^or benzoyl-ATP, are agonists for P_2_Z receptor activation [61]. Zaborina and coworker postulated that the secreted ATP-utilizing enzymes from *P. aeruginosa *convert external ATP to various adenine nucleotides which enhances macrophage cell death through increased P_2_Z receptor activation [54]. Moreover, a mixture of ATP+ADP+AMP+adenosine increased the cell death of peritoneal macrophages in the presence of a clinical isolate of *Burkholderia cepacia *[62]. The work presented here provides evidence that *M. hominis *mediates cell death by both apoptosis and necrosis in HeLa cells, whereas the ecto-ATPase activity of isolated OppA promotes apoptosis only. The findings that the membrane fraction of *M. hominis *depleted of OppA ATPase activity did not provoke death of HeLa cells suggest, that mediated by OppA, one of the degradation products of ATP induces apoptosis. Further studies have to elucidate whether various adenine nucleotides produced by the activity of the ecto-ATPase OppA of *M. hominis *do in fact activate various purinergic receptors thus modulating cell death.

## Conclusion

This is the first description that OppA, the substrate-binding domain of the oligopeptide permease of *Mycoplasma hominis*, induces ATP-release from HeLa cells. ATP hydrolysis by the intrinsic ATPase-activity of OppA results in apoptosis of the host cell which is proposed to guarantee the nutrition uptake and survival of this extra-cellularly colonising pathogen.

## Methods

### Mycoplasma culture, osmotic lysis, and separation of membrane and cytoplasmic proteins

The cultivation of *Mycoplasma hominis *strain FBG, osmotic lyses of the mycoplasma cells as well as the membrane protein preparation with OppA were performed as described previously [35]. For the preparation of the OppA deficient membrane fraction (mem^-^), the membrane fraction was incubated for 8 h with a sepharose-coupled anti-OppA antibody as previously described [33] and the flow through was used. The purity was shown in Western blot analysis demonstrating the absence of OppA and the presence of P50, a surface located protein which was used for quantifying the membrane protein preparations.

### Expression and purification of recombinant proteins

Plasmids pXB and pBX (Roche Applied Science, Mannheim, Germany) were used as expression vectors for the heterogeneous expression of ProteinC-tagged OppA and OppA^ΔP-loop ^(Fig. 1). The plasmids were propagated in *Escherichia coli *SG 13009 (Qiagen, Hilden, Germany) and the recombinant proteins OppA and OppA^ΔP-loop ^were purified as previously described [35].

### HeLa cell culture conditions

HeLa cells were cultured in IMDM supplemented with 10% fetal calf serum and 0.1% penicillin/streptomycin in a humidified atmosphere of 5% CO_2 _in air at 37°C and harvested by dispersing by a 1000 μl pipet and subsequent low speed centrifugation (1200 rpm, 10 min). The proportion of damaged cells was ascertained using trypan blue staining.

### Infection assays

HeLa cells were seeded in 24 well tissue culture plates with 5 × 10^5^cells/ml. After 2 h at 37°C/5%CO_2 _they were incubated with whole *M. hominis *cells (5 × 10^7^cells/ml), the membrane fraction (with (mem+) or without (mem-) OppA) derived from 50 μg mycoplasma lysate, the recombinant OppA protein or the recombinant OppA^ΔP-loop ^mutant [35]. After 2 h unbound cells or proteins were removed by washing twice with medium and the HeLa cells were further cultivated for 1.5, 10, 24, 33 and 48 h. In these infection assays, the mycoplasma samples correspond to approximately 0.5 μg/ml OppA protein for subsequent ATP-measurement and 1 μg/ml OppA for the apoptosis/necrosis assays. To prevent ATP-hydrolysis the supernatant of the HeLa cells was supplemented with 500 μM ATPase inhibitor 4',4', diisothiocyanostilbene 2'2'disulfonic acid (DIDS), 1 h before the mycoplasma protein samples were added. Untreated HeLa cells were used as negative controls.

### ATP-measurement in the culture supernatant

The amount of ATP-release in the supernatant of the different infection assays was measured using the ATP Determination Kit (Molecular Probes, Eugene, Oregon, USA) according to the manufacturer's instructions.

### Measurement of apoptosis and necrosis by flow cytometric analysis

Discrimination between apoptotic and necrotic cells was performed by flow cytometric analysis in using the TACS™ Annexin-V-FITC Kit (Trevigen Inc., Netherlands) and 7-amino-actinomysin D (7-AAD) (BD Biosciences Pharmingen, San Diego, CA) following the manufacturers' instructions.

Briefly, HeLa cells of the infection assays were harvested by repeated rinsing (3 times) in cold phosphate-buffered saline, PBS, pH 7,4 and subsequent low-speed centrifugation (1200 rpm, 10 min). The cell pellets were resuspended at 1 × 10^6 ^cells/ml in binding buffer (10 mM HEPES, pH 7.4, 150 mM NaCl, 5 mM KCl, 1 mM MgCl_2_, 1,8 mM CaCl_2_) and 100 μl fractions were then incubated for 15 min with 125 ng Annexin-V-FITC or 10 min with 250 ng 7-AAD at room temperature in the dark. After the addition of 400 μl binding buffer the samples were analysed by flow cytometry within one hour. As a positive control for apoptotic cells HeLa cells were irradiated with ultraviolet (UV) light of 312 nm wavelength for 2 min. Flow cytometric analysis was performed using an FACS-Calibur and CellQuest software from BD Immunocytometry Systems (San Jose, CA).

### TaqMan PCR for quantifying HeLa cells

HeLa cells of an infection assay were harvested, washed with Tris-buffered saline, TBS (50 mM Tris/HCl, pH 7.5, 100 mM NaCl), sedimented for 10 min at 1200 rpm and resuspended in 100 μl TBS supplemented with Proteinase K (100 μg/ml). The cell samples were incubated for 60 min at 56°C followed by 30 min inactivation of the Proteinase K at 95°C. After a short centrifugation, the cell lysates were ready to be used in PCR. If not tested immediately, they were stored at -20°C.

To determine the HeLa cell count within each sample, the copy numbers of the single-copy house-keeping gene encoding the glyceraldehyde-3-phosphate dehydrogenase (GAPDH) were verified in real-time PCR. The GAPDH-real-time PCR was carried out in a total volume of 25 μl consisting of 1 × Eurogentec MasterMix without ROX, 5 mM MgCl_2_, Amperase, 300 nM each primer: Gap-for (5'-CCA CCC ATG GCA AAT TCC-3') and Gap-rev (5'-ATG GGA TTT CCA TTG ATG ACA AG-3'), 200 nM Gap-probe (5'-FAM-TGG CAC CGT CAA GGC TGA GAA CG-3' TAMRA) and 2,5 μl of the DNA containing cell lysate. Thermal cycling conditions were as follows: 1 cycle at 50°C for 10 min, 1 cycle at 95°C for 10 min followed by 45 cycles at 95°C for 15 sec and 60°C for 1 min. Each sample was analysed in duplicates. Cycling, fluorescent data collection and analysis were carried out with an iCycler from BioRad according to the manufacturer's instructions.

## Authors' contributions

MH carried out all experimental part. MH and BH conceived the study, participated in its design and coordination and drafted the manuscript. Both authors read and approved the final manuscript.
